# A mantle assist for the early geodynamo and planetary shielding

**DOI:** 10.1093/nsr/nwag046

**Published:** 2026-01-23

**Authors:** John A Tarduno

**Affiliations:** Department of Earth & Environmental Sciences, University of Rochester, USA; Department of Physics & Astronomy, University of Rochester, USA; Laboratory for Laser Energetics, University of Rochester, USA

Earth’s magnetic field shields the planet and life from harmful cosmic radiation, so learning more about the key factors that defined dynamo history is central to our understanding of planetary habitability [1,2]. An early core dynamo can be thought of as a slave of the mantle because it controls core–mantle boundary (CMB) heat flux, which in turn governs convection. In the billions of years prior to the onset of inner core growth [3], the mantle should have been the principal governor of the efficiency of the dynamo, determining whether it existed at all. Labrosse and colleagues [4] emphasized this control when they proposed that, early in Earth history, a basal magma ocean (BMO) smothered a core dynamo by preventing the requisite CMB heat flux. But this inference assumed that there would be no processes inherent to the BMO that enhanced this flux. The subsequent collection of paleomagnetic data defining a dynamo as old as 4.2 billion years [1] revealed that the magnetic field was not prohibited as Labrosse and colleagues had suggested, but left unanswered whether the BMO could still have had an important influence on the dynamo.

**Figure 1.**
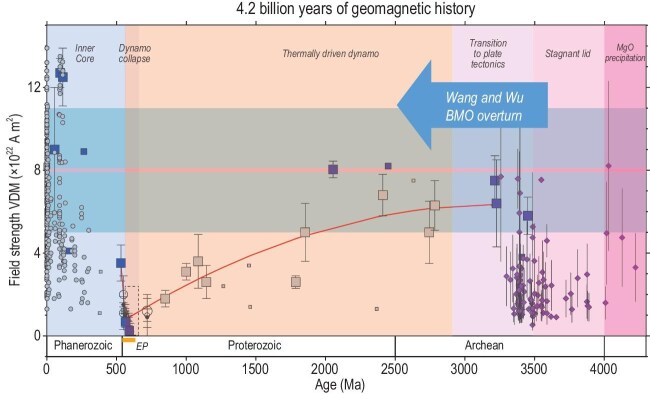
BMO overturn proposed by Wang and Wu [5] shown with geomagnetic field strength history [1] updated with new analyses of the 1-Ga field [10]. Shaded field around the Recent value is a variation of the field strength seen over the last 800 kyr. The trend curve shows the decay of the field leading into the Ediacaran Period (EP). See [1] for data sources and interpretations.

Wang and Wu [5] have taken up this question and studied the potential for mantle overturn induced by water in the BMO. Their models predict high magnetic-field strengths upon this overturn, corresponding to the earliest Paleoarchean to Neoarchean times, roughly 3.5–2.5 billion years ago (Ga). This prediction shows striking correspondence to the paleomagnetic record (Fig. 1). The field strength is subdued from 4.0 to 3.5 Ga, consistently with a stagnant-lid Earth [6], and then increases to strong, present-day values [1,7] after 3.5 Ga that persisted throughout to the Proterozoic [8].

This is a remarkable first-order agreement between model predictions and observations, but there is also a mismatch. The exquisite paleointensity data of Wentao Huang [9] define a strong field at ∼2 Ga, suggesting that the high-field-intensity period was longer than predicted by BMO overturn alone. As recognized by Wang and Wu [5], the establishment of truly global plate tectonics would also have increased the CMB heat flux and dynamo strength in Neoarchean to Proterozoic times [1, 5]. Parsing out these contributions, as well as improving upon the still sparse paleomagnetic record, are promising directions for future research.

Wang and Wu [5] do not consider a dynamo generated in the BMO and, for Earth, this appears to be excluded by considerations of thermal conductivity [9]. But BMOs are expected for other terrestrial planets in our solar system, including Mars, and for exoplanets. Whether BMO overturn occurs will depend on initial conditions and is another area that is ripe for future investigation. The Wang and Wu [5] work highlights that, if overturn occurs, then BMOs should be added to the list of features that can assist—rather than diminish—the dynamo, ultimately leading to magnetic shielding that enhances planetary habitability.
